# Epigenetic regulation of pancreatic adenocarcinoma in the era of cancer immunotherapy

**DOI:** 10.1007/s00535-022-01915-2

**Published:** 2022-09-01

**Authors:** Kazumichi Kawakubo, Carlos Fernandez-del Castillo, Andrew Scott Liss

**Affiliations:** 1 Department of Gastroenterology and Hepatology, Faculty of Medicine and Graduate School of Medicine, Hokkaido University, Sapporo, Japan; 2 Department of Surgery, Massachusetts General Hospital, Harvard Medical School, Boston, MA USA

**Keywords:** Pancreatic adenocarcinoma, Epigenetics, Immune checkpoint blockade

## Abstract

Pancreatic adenocarcinoma is a lethal cancer with poor response to chemotherapy and immune checkpoint inhibitors. Recent studies suggest that epigenetic alterations contribute to its aggressive biology and the tumor microenvironment which render it unresponsive to immune checkpoint blockade. Here, we review our current understandings of epigenetic dysregulation in pancreatic adenocarcinoma, its effect on the tumor immune microenvironment, and the potential for epigenetic therapy to be combined with immune checkpoint inhibitors.

## Introduction

Pancreatic adenocarcinoma (PDAC) is highly fatal malignancy with a 5-year survival rate of less than 10% and is the 4th leading cause of cancer-related death in USA [1, 2]. While surgical resection is the only cure for PDAC, 80% of patients are diagnosed with advanced-stage disease and are not eligible for surgery [3]. Recent genomic analysis facilitated the sub-classification of pancreatic adenocarcinoma based on molecular signatures; however, the use of most of molecular targeted therapies has failed to show clinical benefit to date [4–7]. Epigenetic regulation of gene expression includes the post-translational modification of histone proteins and methylation of DNA. These modifications are controlled by enzymes and adaptor proteins that are referred to as epigenetic ‘writers’, ‘erasers’, and ‘readers’ [8]. Dysregulation of these proteins contributes to carcinogenesis and pathobiology, including pancreatic cancer [9, 10]. These epigenetic regulators are getting attention as therapeutic targets for cancer therapy.

Immune checkpoint inhibitors targeting cytotoxic T-lymphocyte-associated protein 4 (CTLA4) and programmed cell death 1/programmed death ligand 1 (PD1/PDL1) have been used with great success in several cancers and are now widely accepted as one of the standard therapies in cancer treatment [11]. Cancers with high tumor mutation burden and mismatch repair deficiency are associated with favorable response to immune checkpoint inhibitors, but their rates are less than 1% in pancreatic adenocarcinoma [12–14]. Moreover, the tumor microenvironment in pancreatic adenocarcinoma is characterized by abundant fibrosis and cancer-associated fibroblast, which contribute to the immune evasion and immunotherapy failure [15–17]. However, the precise mechanism of resistance is not fully understood in pancreatic cancer.

Several epigenetic modifiers affect the tumor immune microenvironment and are being investigated as anticancer treatment in combination with immune checkpoint inhibitors [18]. In this review article, we summarize the epigenetic drug therapies that affect tumor immunity against pancreatic cancer.

## DNA methylation

DNA methylation regulates gene expression and chromatin structure, which is mainly controlled by DNA methylation writers (DNA methyltransferase; DNMT family) and erasers (ten–eleven translocation methylcytosine dioxygenase; TET family). The genes encoding DNMT and TET family members are frequently mutated in cancers including pancreatic cancer [19]. DNMT3a and DNMT3b promote de novo DNA methylation during embryogenesis and germ cell development, whereas DNMT1 is required for the maintenance of DNA methylation during cell replication [20, 21]. Aberrant focal DNA hypomethylation by loss of function mutation in DNMT3a promotes hematologic malignancies and is associated with poor prognosis [22, 23]. In addition, DNMT family members are more highly expressed in cancer cells than that in normal cells [24, 25] and the resulting aberrant DNA hypermethylation contributes to pancreatic cancer development [26, 27].

DNA methyltransferase (DNMT) inhibitors are the most commonly used epigenetic drug for cancer treatment. These inhibitors are nucleoside cytidine analogues [5-Azacitidine (5-Aza), 5-aza-2′-deoxycytidine (decitabine), and SGI-110 (guadecitabine)] that are incorporated into DNA and bind DNMTs, resulting in reactivation of the genes silenced by aberrant methylation by DNMTs [28]. In pancreatic cancer cell lines with low DNMT1 expression, the DNMT inhibitor decitabine depletes DNMT1 and exerts anti-tumor effects [25]. While guadecitabine sensitizes pancreatic cancer cells to chemotherapeutic agents [29], there is only one phase I study evaluating the anti-tumor effect of DNMT inhibitor on pancreatic cancer [30].

The ten–eleven translocation (TET) family of α-ketoglutarate-dependent dioxygenases indirectly promotes DNA demethylation by hydroxylation of 5-methylcytocine to 5-Hydroxymethylcytocine [31]. Characterization of pancreatic pre-cancerous lesions from pancreatic intraepithelial neoplasia, intraductal papillary mucinous neoplasms, intraductal event during pancreatic oncocytic papillary neoplasms, and mucinous cystic neoplasms by immunohistochemistry revealed that downregulation of 5-hydroxymethylcytocine and TET1 is an early carcinogenesis [32]. Moreover, CRISPR-Cas9-induced knockout of TET1 showed that TET1 suppress pancreatic cancer progression via inhibition of the Wnt pathway [33]. Taken together, these studies demonstrate that increased DNA methylation, either by TET inhibition or DNMT activation, has an important role for pancreatic cancer development.

Isocitrate dehydrogenase (IDH) enzymes IDH1 and IDH2 are frequently mutated in several cancers and a gain-of-function activity promotes conversion of α-ketoglutarate (α-KG) to the oncometabolite 2-Hydroxyglutarate (2-HG) [34, 35]. The accumulation of 2-HG inhibits α-ketoglutarate-dependent dioxygenases including TET family. Subsequently, IDH-mutated cancers have global DNA hypermethylation leading to an epigenetic gene silencing [36, 37]. A recent study in glioma revealed that IDH mutations are associated with decreased PD-L1 expression via hypermethylation of the STAT1 promoter and impaired IFN-γ signaling [38]. Employing a mouse model of IDH-mutant glioma, the authors demonstrated that selective inhibition of mutant IDH suppresses 2-HG production, restoring T-cell immunity and enhancing the anti-tumor effect of immune checkpoint inhibitor. Mutation and amplification of IDH are not common in pancreatic cancer, but the relationship between IDH inhibition and immune checkpoint blockade remains to be clarified.

There is little study to evaluate the combination therapy of immune checkpoint inhibitors and DNA demethylating agents for pancreatic cancer. However, recent studies revealed that DNA demethylation increase tumor antigen and reactivate endogenous retroviral sequences [39, 40]. In terms of relationship to immune checkpoint inhibitor, the DNMT inhibitor 5-azacytidine inhibits immune evasion in cancer through up-regulation of tumor antigen presentation and T-cell chemokine expression [41, 42]. Interestingly, decitabine improve the effectiveness of immune checkpoint inhibitor via modulation of stroma-rich tumor microenvironment by inducing STAT1 and IFN-γ signaling [43, 44]. These findings facilitate the ongoing study of DNMT inhibitor combined with immune checkpoint inhibitors for pancreatic cancer.

## Histone acetylation

Gene expression regulated by histone acetylation is controlled by writers; histone acetyltransferase (HATs), and erasers; histone deacetylases (HDACs) [45]. Histone acetylation is associated with open chromatin structure and promotes gene expression. HDAC inhibitors exert anti-tumor effect by re-expression of silenced genes by promoting histone acetylation [46].

In humans, there are 18 HDACs grouped into four classes (class I, II, III, and IV) based on their homology to yeast HDAC. There are several HDAC inhibitors approved by FDA [47]. In preclinical models, class I HDAC and BET-inhibitors (discussed below) showed synergistic anti-tumor effects against pancreatic cancer through the dysregulation of FOSL-1 [48]. A class II HDAC inhibitor combined with the proteasome inhibitor carfilzomib induces cell cycle arrest in pancreatic cancer through FOXO3a activation [49]. Moreover, there is growing evidence that modulating histone acetylation can impact the immune microenvironment. HDAC3 regulates tumor immunity by increasing STAT3 signaling and PDL1 expression [50]. Trichostatin-A, a class I and II inhibitor, suppresses M2 macrophage polarization and MDSCs infiltration in syngeneic cancer mouse models [51]. Entinostat, class I HDAC inhibitor, modulates immune-resistant pancreatic cancer microenvironment by suppressing the myeloid-derived suppressor cells (MDSCs) [52].

Efficacy of HDAC inhibitors was well established in hematologic malignancies, but most clinical trials failed to demonstrate clinical benefit as a single agent in solid cancers, including pancreatic cancer [53]. For solid tumors, the anticancer effect of HDAC inhibition is dependent on the tumor immune microenvironment [54]. In melanoma, HDAC inhibition upregulates PDL1 expression and enhances the anti-tumor effect of immune checkpoint inhibitor [55]. While there have been several phase I studies evaluating the combination therapy with HDAC inhibitor [30, 56–64], phase II trials have not yet revealed clinical benefit. In a phase II trial, an oral HDAC inhibitor, CI994 (tacedinaline), combined with gemcitabine did not improve overall survival in pancreatic cancer as compared to gemcitabine alone [65]. Another phase II study demonstrated that combination therapy of panobinostat and bortezomib provided a modest median progression free survival of 2.1 months in patients with pancreatic cancer and was not recommended [66].

HATs have become one of the top targets of cancer immunotherapy. HATs are composed of CREB-binding protein (CBP) and its homologue E1A-associated protein p300 (P300) and augment cancer immunotherapy through induction of MHC-I antigen presentation [67]. On the other hand, P300 acetyltransferase regulates the expression of PDL1 on plasma membrane by acetylating the cytoplasmic domain and prevents the evasion of immune surveillance via nuclear translocation [68]. HAT1 is overexpressed in PDAC patients and associated with poor prognosis. Moreover, HAT1 inhibition improved the immune checkpoint blockade treatment by decreasing the PDL1 expression in PDAC xenograft model via cell-intrinsic signals, including the regulation of autophagy and the mTOR pathway [69]. The effect of HAT1 on tumor immunotherapy remains unrevealed.

Bromodomain and extraterminal (BET) family members are ‘readers’ of acetylated lysine residue on histones. In pancreatic cancer cell lines, BET proteins promote cancer progression, at least in part, by interaction with GLI transcription factors as well as impacting the tumor microenvironment by regulating the expression of SHH and IL6 [70, 71]. Moreover, BET proteins play a key role in mediating the PDAC-cancer-associated fibroblast (CAF) cross-talk that is required for the formation of PDAC matrisome [72]. BET bromodomain inhibitor JQ1 combined with HDAC inhibitor SAHA demonstrated synergistic antiproliferative effect by de-repression of p57 [73]. BET proteins induce PDL1 expression in several cancers by binding to its promoter and enhancer [74]. JQ1 combined with PD-1 blockade enhances the anti-tumor response in Kras-mutant lung cancer by reducing the T-reg and increasing T-cell infiltration with T-helper type 1 cytokine [75]. In pancreatic cancer, a dual inhibitor of BET proteins and HATs suppress KRAS/MAPK signaling and augment PDL1 blockade through recruitment of cytotoxic T-cell [76]. Several BET bromodomain inhibitors are vigorously investigated in clinical trials of anticancer treatment and only one phase I study is evaluating the combination therapy of BET inhibitor and immune checkpoint inhibitor [77, 78]. However, these studies highlight that epigenetic silencing by BET protein inhibitors promotes anti-tumor immunity and potentiates anti-tumor effect of immune checkpoint inhibitor in pancreatic cancer.

## Histone methylation

The effect of post-translational histone methylation on gene expression depends on the methylation status of specific histone tail residues [79]. For example, histone H3 trimethylated at lysine 4 (H3K4me3) promotes gene expression, while H3K27me3 suppresses it [80]. Histone methylation is regulated by histone methyltransferases (HMTs, writers) and demethylases (HDMs, erasers). Histone methylation is dysregulated in many cancers [81]. Low levels of H3K4me2, H3K18ac, and H3K27me3 are associated with poor survival in pancreatic cancer [82, 83]. Mutation in histone proteins can also have an oncogenic effect [84]. One of the mutations, H2BG53D (termed oncohistone), is associated with the progression of pancreatic cancer [85, 86]. However, the effect of oncohistone mutation on pancreatic cancer progression is not well elucidated.

HMTs are being investigated as a therapeutic target for several cancers. Enhancer of zeste homologue 2 (EZH2) is one of the most widely investigated HMTs and is responsible for the formation of H3K27me3. EZH2 promotes the progression of several cancer [87] and promotes cell migration and invasion of pancreatic cancer cell lines [88]. EZH2 is one of four core subunits of Polycomb repressive complex 2 (PRC2), which maintains bivalency at the promoters of MHC-1 antigen processing pathway gene to silence MHC-1 expression and promote evasion of T-cell-mediated immunity. Pharmacological inhibition of EZH2 reverses PRC2-mediated MHC-1 silencing by suppressing the repressive H3K27me3, leading to re-establishment of effective T-cell-mediated anti-tumor immunity [89]. In head and neck cancer cell lines, EZH2-inhibitors (GSK126 and EPZ6438) enhance antigen presentation and overcome immune checkpoint resistance [90]. EZH2 is also associated with silencing of TH-1 type chemokines, including CXCL9 and CXCL10 [91]. GSK126 combined with a DNMT inhibitor (GSK-J4) epigenetically reprogrammed the tumor immune microenvironment by restoring the expression of these chemokines. There is only one phase I study evaluating EZH2-inhibitors combined with immune checkpoint blockade for a solid tumor including melanoma, lung cancer, and renal cell carcinoma; however, targeting the epigenetic silencing of antigen presentation is an attractive mechanism to induce tumor immunity in pancreatic cancer.

SET domain bifurcated 1 (SETDB1) catalyzes the di- and trimethylation of H3K9 (H3K9me3) to switch from euchromatic to heterochromatic states. Aberrant SETDB1 expression is observed in various cancers and is associated with carcinogenesis [92]. In pancreatic cancer, SETDB1 inhibits apoptosis by regulating p53 expression [93]. A recent CRISPR-Cas9 chromatin regulator screen revealed that SETDB1 plays an important role for suppressing tumor-intrinsic immunogenicity and its amplification is associated with immune evasion and resistance to immune checkpoint inhibitors [94]. CRISPR-induced knockdown of SETDB1 in lung and melanoma cell lines induces transposable elements-encoded viral antigens and triggers cytotoxic T-cell response. While there is no SETDB1-specific methyltransferase inhibitor available, these preclinical studies suggest that it might be a therapeutic target of cancer immunotherapy for pancreatic cancer.

Disruptor of telomeric silencing 1-like (DOT1L) is a histone H3 lysine 79 methyltransferase. DOT1L is well investigated in prostate cancer and MLL-fusion leukemia [95, 96]. Decreased DOT1L along with low levels of H3K79me3 is associated with epithelial–mesenchymal transition (EMT) and PDL1 expression, but the effect of DOT1L on tumor immunity is still under investigation [97].

There are additional studies suggesting the promise of other histone methyltransferases as candidates for drug development to enhance tumor immunity. Nuclear receptor-binding SET domain protein 1 (NSD1) is a histone H3 lysine 36 methyltransferase. NSD1 mutations are observed in head and neck cancer and are associated with increased PDL1 expression [98, 99]. Knockdown of NSD1 in head and neck cancer cells resulted in decreased expression of CCL5 and suppressed T-cell infiltration into the tumor microenvironment, suggesting that NSD1 inactivation in cancer has implications for cancer immunotherapy [100]. In pancreatic cancer, high NSD1 expression is associated with poor prognosis [101]. Histone-lysine N-methyltransferase (KMT2) family catalyze the formation of trimethylation of H3K4 (H3K4me3). KMT2s are frequently mutated especially in hematologic malignancies and are associated with favorable response to cancer immunotherapy [102, 103]. KMT2A is highly expressed in pancreatic cancer cells followed by H3K4me3-mediated PD-L1 expression and immune evasion [104, 105]. There is no FDA approved drug to inhibit NSD1or KMT2 family members, but inhibition of these enzymes combined with immune checkpoint blockade may be promising strategies.

Unlike the HMTs described above, protein arginine methyltransferases (PRMTs) catalyze the methylation of arginine residues of histones. PRMTs are classified into three enzymes (type I, II and III). PRMT5 (type II PRMT) promotes EMT and PRMT1 (type I PRMT) is associated with poor prognosis in pancreatic cancer [106, 107]. Recent work revealed that inhibition of PRMTs promotes anti-tumor immunity by modulating RNA splicing [108]. Type 1 and 2 PRMT inhibition promotes T-cell-mediated anti-tumor immunity [109, 110]. In a xenograft model of mouse pancreatic cancer, a type I PRMT inhibitor combined with PD-L1 blockade demonstrated anti-tumor effect through increasing infiltration of CD8 + T cells [111].

Histone demethylases are also being investigated as epigenetic regulators of pancreatic cancer. Lysine-specific histone demethylase (KDM) family members remove methylated lysine residue of histone tails. KDM1A maintains pancreatic cancer progression through HIF1α-dependent glycolysis [112]. In a subcutaneous synergistic mouse tumor model, KDM1A is a potent inhibitor of anti-tumor immunity, and its ablation activates the type I interferon along with CD8 + T-cell infiltration [33, 113]. High expression of KDM5A is associated with improved response to immune checkpoint blockade by enhancing TLR signaling in several mouse models of cancer, including breast cancer, melanoma and fibrosarcoma [114]. KDM5C mutations predict the favorable outcome of immune checkpoint inhibitor by pan-cancer data base analysis [115]. KDM4A inhibition activates anti-tumor immunity and enhances anti-PD1 immunotherapy in squamous cell carcinoma [116]. Interestingly, KDM2A expression in cancer-associated fibroblast promotes carcinogenesis and inhibition of KDM2A suppress the tumor growth via inhibiting PDL1 expression in breast cancer fibroblast cell lines [117]. Recent large data base analysis revealed that up-regulation of KDM5A/B/C expression was associated with infiltrating immune cells in pancreatic cancer [118]. Since the activity of KDM family members regulate tumor immunity, therapeutically targeting these proteins in combination with immune checkpoint inhibitors against pancreatic adenocarcinoma needs investigation.

In summary, several epigenetic drugs can augment the tumor immunity in pancreatic adenocarcinoma. There is still a long way to go before these therapies become a standard treatment in the daily practice. Considering the unique tumor microenvironment of PDAC characterized by abundant non-cancer components that create additional barriers to immune checkpoint inhibitors, therapies that target the tumor microenvironment might enhance the effect of epigenetic drugs on cancer immunotherapy. Table 1 summarizes the current clinical trial combining epigenetic therapy with immune checkpoint inhibitors for pancreatic cancer. The improvement in specificity of current epigenetic drugs will provide further possibilities for combination therapies. Collectively, epigenetic therapy combined with immune checkpoint blockade turns immune cold into hot tumor microenvironment and is promising strategy for the treatment of pancreatic adenocarcinoma.

**Table 1:** Clinical trials of epigenetic therapies with immune checkpoint inhibitors

NCT number		Immune checkpoint inhibitors	Status	Phases
DNMT inhibitors				
NCT03264404	Azacytidine	Pembrolizumab (Anti-PD1)	Active, not recruiting	Phase2
NCT03257761	Guadecitabine	Durvalumab (Anti-PDL1)	Active, not recruiting	Phase1
HDAC inhibitors				
NCT03250273	Entinostat	Nivolumab (Anti-PD1)	Completed	Phase2
NCT02909452	Entinostat	Pembrolizumab (Anti-PD1)	Completed	Phase1
BET-inhibitors				
NCT02419417	BMS-986158	Nivolumab (Anti-PD1)	Completed	Phase1/2
EZH2-inhibitors				
NCT03525795	CPI-1205	Ipilimumab (Anti-CTLA4)	Completed	Phase 1/2

## Abbreviations

PD1: Programmed cell death 1
PDL1: Programmed death ligand 1
CTLA4: Cytotoxic T-lymphocyte-associated protein 4
DNMT: DNA methyltransferase
HDAC: Histone deacetylase
HMT: Histone methyltransferase
HAT: Histone acetyltransferase
TET: Ten–eleven translocation
