# Correction to: Epigenetic regulation of pancreatic adenocarcinoma in the era of cancer immunotherapy

**DOI:** 10.1007/s00535-022-01922-3

**Published:** 2022-09-17

**Authors:** Kazumichi Kawakubo, Carlos Fernández-del Castillo, Andrew Scott Liss

**Affiliations:** 1 Department of Gastroenterology and Hepatology, Faculty of Medicine and Graduate School of Medicine, Hokkaido University, Sapporo, Japan; 2 Department of Surgery, Massachusetts General Hospital, Harvard Medical School, Boston, MA USA

## Correction to: J Gastroenterol 10.1007/s00535-022-01915-2

In the original publication of the article the second author name should be “Carlos Fernández-del Castillo” and not “Carlos Fernandez-del Castillo”.

